# Treatment of Refractory Mucosal Leishmaniasis Is Associated with Parasite Overexpression of HSP70 and ATPase and Reduced Host Hydrogen Peroxide Production (Brief Report)

**DOI:** 10.3390/biomedicines12102227

**Published:** 2024-09-30

**Authors:** Ada Amália Ayala Urdapilleta, Adriana de Oliveira Santos Alfani, Daniel Holanda Barroso, Felipe Vinecky, Suzana da Glória Amaral Vaz Bandeira, Alan Carvalho Andrade, Jorge Alex Taquita, Izabela Marques Dourado Bastos, Raimunda Nonata Ribeiro Sampaio

**Affiliations:** 1 Post-Graduation Program in Clinical Medicine (PPGCM), Faculty of Medicine (FM), Campus Universitário Darcy Ribeiro, University of Brasília (UnB), UnB Área 1—Asa Norte, Brasilia 70910-900, DF, Brazil; ada.urdapilleta@gmail.com (A.A.A.U.); 1758455@etfbsb.edu.br (A.d.O.S.A.); tinamy.447@hotmail.com (S.d.G.A.V.B.); rnrsampaio@hotmail.com (R.N.R.S.); 2 Federal Institute of Brasília, Brasília 70910-900, DF, Brazil; 3 Dermatology Service, University Hospital of Brasília, Faculty of Medicine, University of Brasília (UnB), Brasilia 70910-900, DF, Brazil; 4 Dermatomycology Laboratory, School of Medicine, University of Brasília (UnB), Brasilia 70910-900, DF, Brazil; 5 Post-Graduation Program, Embrapa Cenargen, Brasilia 70910-900, DF, Braziljorge.melo@embrapa.br (J.A.T.); 6 Molecular Genetics Laboratory (LGM-NTBio), Embrapa, Cenargen, Brasilia 70910-900, DF, Brazil; alan.andrade@ufla.br; 7 Embrapa Recursos Genéticos e Biotecnologia, Brasilia 70910-900, DF, Brazil; 8 Pathogen–Interaction Laboratory, Department of Cell Biology, Institute of Biology, University of Brasília (UnB), Brasilia 70910-900, DF, Brazil; dourado@unb.br; 9 Post-Graduation Program in Health Sciences (PGHC), Faculty of Health Sciences, University of Brasilia (UnB), Brasilia 70910-900, DF, Brazil

**Keywords:** mucosal leishmaniasis, *Leishmania* (*V.*) *braziliensis*, drug resistance, putative heat shock protein 70 (hsp70), hydrogen peroxide (H_2_O_2_), therapeutic failure

## Abstract

Background: Mucosal leishmaniasis (ML) is a deforming type of American Tegumentary Leishmaniasis caused by *Leishmania* (*Viannia*) *braziliensis* that frequently does not respond to treatment. Despite its relapsing clinical course, few parasites are usually found in mucosal lesions. Host and parasite factors may be responsible for this paradox in the pathogenesis of the disease, allowing for both a low parasite burden and the inability of the host to clear and eliminate the disease. Methods and results: In this work, we present a clinical case of relapsing ML that was treated for 25 years without success with SbV, N-methyl glucamine, sodium stibogluconate, amphotericin B deoxycholate, gabromycin, antimonial plus thalidomide, liposomal amphotericin B, Leishvacin (a vaccine made in Brazil) and miltefosine. In a comparative analysis using nanoscale liquid chromatography coupled with tandem mass spectrometry of protein extracts of *L.* (*V.*) *braziliensis* promastigotes isolated from the patient and from the reference strain (MHOM/BR/94/M15176), we observed increases in ATPase and HSP70 protein levels in the parasite. We also observed an impairment in the production of hydrogen peroxide by peripheral mononuclear blood monocytes (PBMCs), as assessed by the horseradish peroxidase-dependent oxidation of phenol red. Conclusions: We hypothesise that these parasite molecules may be linked to the impairment of host parasiticidal responses, resulting in Leishmania persistence in ML patients.

## 1. Introduction

Leishmaniasis is a major public health problem worldwide, with an estimated 700,000 to 1,000,000 new cases annually [1]. Mucosal leishmaniasis (ML) is one of the most serious manifestations of American cutaneous leishmaniasis (ACL). It does not seem to heal spontaneously and can cause the destruction of nasal cartilage and soft tissue, with possible facial disfiguration and even mortality [2,3,4].

Treatment failure is a growing problem, and parasitic resistance is the main underlying factor [5]. The proportion of patients with the disease one year after treatment was 30%, according to a retrospective Brazilian hospital study [6,7]. The identification of parasite pathogenic molecules is scientifically valuable since it allows the development of new targeted drugs, but it can also have an immediate clinical impact through its prognostic value in predicting resistance and susceptibility [8,9]. Proteomic and transcriptomic studies of the parasite have suggested possible factors associated with this resistance to treatment, including proteins involved in oxidative stress [9,10] and metabolism [11].

Here, we wanted to explore the possible physiopathological causes of treatment failure in a patient diagnosed with ML caused by *Leishmania* (*V.*) *braziliensis* who had undergone multiple treatments over the course of 25 years. To achieve this goal, we compared the parasites cultured from the patient with those from a control strain subjected to treatment. We measured the expression of proteins to identify molecules potentially involved in drug resistance and assessed hydrogen peroxide production in monocytes from this patient.

## 2. Materials and Methods

### 2.1. Culture

The control strain (*Leishmania* (*V.*) *braziliensis*-MHOM/BR/94/M15176) used in this study was provided by the Evandro Chagas Institute, and the experimental strain was collected from a patient in 2009 and cryopreserved in liquid nitrogen in the laboratory until analysis one and a half years later. At the time of sample collection, the patient had active mucosal lesions. Before analysis, both strains were decryopreserved and injected into the feet of C57BL/6 mice, and after the appearance of lesions, the parasites were collected with a 1 mL syringe containing 0.5 mL of 0.9% saline, cultured for five days in McNeal, Novy and Nicolle (NNN) media supplemented with FBS at 22 °C, and then seeded in Schneider medium supplemented with 20% FBS and gentamicin, as described elsewhere [12]. After an initial growth phase of one week, parasite numbers were measured in a Neubauer chamber daily according to the methodology proposed by Brener [13].

### 2.2. Mass Spectrometry Analysis

#### 2.2.1. Protein Extraction

Protein extraction was performed according to the protocol proposed by Sussulini [14], with modifications. Briefly, a parasite culture suspension in the logarithmic growth phase was centrifuged at 1000× *g* at 4 °C for 10 min. After three washes with ice-cold PBS, the parasite pellet was solubilized in 1 mL of petroleum ether for 15 min under strong agitation to remove lipids. The proteins were then extracted with 1 mL of 50 mM Tris-HCl (pH 8.8) containing 1.5 mM potassium chloride, 10 mM dithiothreitol-DTT, 1.0 mM phenylmethylsulfonyl fluoride (PMSF), and 0.1% sodium dodecyl sulfate (SDS) (*w*/*v*) under shaking for 10 min in an ice bath. After centrifugation, the supernatant (protein extract) was collected, and the proteins were precipitated with acetone overnight. The proteins were quantified via a Qubit™ protein assay kit (Invitrogen, Waltham, MA, USA).

#### 2.2.2. Protein Digestion

The samples were subsequently resuspended in 60 µL of 50 mM sodium bicarbonate. Then, 25 µL of 0.2% RapiGest SF solution (Sigma–Aldrich, St. Louis, MO, USA) was added and vortexed. The mixture was then heated for 15 min at 80 °C and centrifuged. The proteins were reduced by adding 2.5 µL of 100 mM DTT and heating at 60 °C for 30 min. The samples were cooled to room temperature, alkylated with 2.5 µL of 300 mM iodoacetamide, incubated at room temperature in the dark for 30 min, and finally trypsinized with 10 µL of 50 mM trypsin (Madison, WI, USA) for 20 h at 37 °C. The hydrolysis reaction was stopped by the addition of 10 µL of 5% trifluoroacetic acid (TFA), and the samples were incubated at 37 °C for 90 min. The samples were then centrifuged at 14,000 rpm and 6 °C for 30 min at 5425R in a microcentrifuge (Eppendorf, Hamburg, Germany). The supernatant was transferred to a flask, and 5 µL of alcohol dehydrogenase-ADH (1 pmol/µL) and 85 µL of 3% acetonitrile containing 0.1% formic acid were added. The final protein concentration was 250 ng/µL, the ADH concentration was 25 fmol/µL, and the final volume was 200 µL. The full protocol used for protein digestion is presented in the Supplementary Material.

#### 2.2.3. Protein Identification

The resulting peptide mixture was analysed via high-performance liquid chromatography as described by Silva et al [15,16], with modifications as follows. The experiments were performed in reversed-phase mode via a capillary column (NanoAcquity, UPLC-Waters, Milford, MA, USA) with a gradient of 2–97% B (AcN + 0.1% formic acid). The data obtained from the NanoLC-MS^E^ experiments were processed via the ProteinLynx program (version 2.0) from Waters/Micromass (Milford, MA, USA), an application for identifying proteins via a database obtained from the National Center for Biotechnology Information (NCBI). NanoLC-MSE experiments were performed in duplicate, but processing in the ProteinLynx software was performed only once. The analysis of data collected by mass spectrometry of the tryptic peptides and the full protocol for identification are provided in the Supplementary Material (Supplementary Tables S1 and S2).

### 2.3. Hydrogen Peroxide Production

Hydrogen peroxide (H_2_O_2_) production by peripheral mononuclear blood monocytes (PBMCs) before the last treatment was assessed by horseradish peroxidase-dependent oxidation of phenol red, as follows [17]. Triplicate samples, each containing 1.5 × 10^5^ cells, were seeded into 96-well plastic microplates (Corning, NY, USA) containing RPMI 1640. Following a 1 h incubation with a solution composed of 5.5 mM dextrose, 0.5 mM phenol red, and 19 U/mL of horseradish peroxidase type I RZ 1.0 (Sigma–Aldrich, San Luis, MO, USA), with or without the addition of 2 μM phorbol 12-myristate 13-acetate 4-O methyl ether (Sigma–Aldrich, St. Louis, MO, USA), in the presence or absence of 40 μg/L pravastatin sodium, the enzymatic reaction was halted by the addition of 10 μL of 1 N NaOH to each well. The absorbance was then measured at 620 nm via a Multiskan Titertek microplate reader. The average value of the triplicate samples was calculated for each sample and expressed as the optical density (OD) for 1.5 × 10^5^ PBMCs per hour. Consistent results were obtained across the triplicate observations.

## 3. Results and Discussion

### Clinical Case

**Figure 1:**
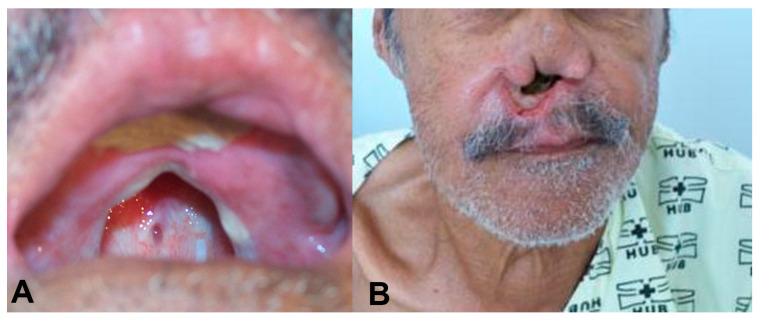
(**A**) An oral lesion with destruction of the soft and hard palate. The patient used a palatal prosthesis to allow oral feeding. (**B**) Complete destruction of the nasal septum.

Case: In 1987, a 69-year-old male presented with nasal obstruction, rhinorrhea, dysphagia, and hoarseness. In infancy, he had developed an ulcer in his left leg, with spontaneous healing occurring after approximately four months. Physical examination revealed nasal septum ulcers and infiltration on his lips (Figure 1). Complementary exams, as described by Gomes [18], revealed that the intradermal reaction of the Montenegro (IDRM) area was 6 × 7 mm. Histopathological examination of the skin lesions revealed the presence of lymph-histioplasmacytic dermal infiltrate, and the indirect immunofluorescence ratio for *Leishmania* was between 1:40 and 1:320 during follow-up. The parasite was identified as *Leishmania* (*V.*) *braziliensis* by isoenzymes and PCR according to the routine care of patients with ATL in the service [19,20,21]. Identification by sequencing of the ITS1 region was also performed in this case (Supplementary Material). Direct examination and culture for fungal infections were negative. The presence of clinical lesions or amastigote forms on direct examination of skin lesions or after inoculation in hamsters was used to define recurrence during the follow-up period.

The patient was initially treated with 20 mg SbV/day for 30 days. After an initial improvement, worsening of the disease was observed. We then treated this patient with a variety of drugs, isolated and in combination, including N-methyl glucamine, sodium stibogluconate, amphotericin B deoxycholate, gabromycin, antimonial plus thalidomide, liposomal amphotericin B, Leishvacin [22] (a vaccine made in Brazil), and miltefosine (Table 1). According to the last systematic review on ATL treatment, there is a dearth of evidence on the treatment of ML precluding any conclusion on the comparative results of different treatment modalities [23]. The treatment strategy adopted had to be based in scarce data available on the treatment of ML. Most of the drugs used had already been tested on humans, with variable success, including itraconazole [24,25], mitefosine [26], allopurinol [24,27], sodium stibogluconate [27], amphotericin B [28], pentamidine [29], pentoxifylline [30], and thalidomide [31]. We also tried immunotherapy, which has previously been shown to reduce treatment duration and side effects when combined with antimonials [32]. On multiple occasions, we used combination therapy, which is believed to reduce the risk of drug resistance [33]. Despite that, the lesions gradually worsened, resulting in septal and soft palate perforation and pharynx infiltration with partial obstruction and shortness of breath. The patient gradually developed drilling of the soft palate, resulting in multiple secondary bacterial and fungal infections with feeding problems, leading to the need for gastrostomy and a palatal prosthesis (Figure 1) to improve nutrition and prevent further complications. Miltefosine treatment [34] at 2 mg/kg/day for 42 days resulted in a clinical cure, weight gain (10% of body weight), negativity of indirect immunofluorescence (IIF), and smear and culture results associated with a regression of histopathological infiltrates [35].

However, disease relapse was observed after 2½ years, as demonstrated by intense histopathological staining of the infiltrate and detection of the parasite in culture and after hamster inoculation. After more than 25 years of follow-up and multiple therapeutic regimens, none of the main drugs used for leishmaniasis [36] or various experimental combinations of drugs were successful in curing this patient. The patient died in 2012 from pancreatic cancer while he still had active mucosal lesions.

To better understand the possible mechanisms behind the treatment failure observed in this patient, we studied patient and parasite factors possibly related to treatment outcome. Samples were collected during active disease and after the last treatment had been performed.

The patient showed a basal production of 18.9 µM H_2_O_2_/1.5 × 10^5^ monocytes/h, which decreased to 8.4 µM H_2_O_2_/1.5 × 10^5^ monocytes/h after stimulation with PMA. These values were significantly lower than the production observed in individuals with New World human cutaneous leishmaniasis (146 µM H_2_O_2_/1.5 × 10^5^ monocytes/h) and similar to those reported in noninfected healthy individuals (37 µM H_2_O_2_/1.5 × 10^5^ monocytes/h) [37].

The increase in possible pathogenic molecules in the studied *Leishmania* strain was coupled with a much lower in vitro production of H_2_O_2_ by the patient’s monocytes. H_2_O_2_ is a strong microbicidal molecule produced by phagocytes and is involved in immune defence against *Leishmania*. After phagocytosis of *Leishmania*, macrophages trigger microbicidal mechanisms such as nitric oxide and oxygen radical production. The oxygen radical might have direct effects on parasites, causing protein denaturation and damage to the cell membrane and DNA. An important mechanism by which H_2_O_2_ eliminates microorganisms is the irreversible oxidation of essential proteins such as F0F1 mitochondrial ATPase [38]. The production of oxygen radicals with the disruption of the parasite’s mitochondrial membrane potential is one of the cornerstones of the host’s immune response against *Leishmania* and is also important during the treatment response [39,40]. Thus, it is not surprising that this patient’s lack of response to treatment was associated with a diminished production of hydrogen peroxide.

**Figure 2:**
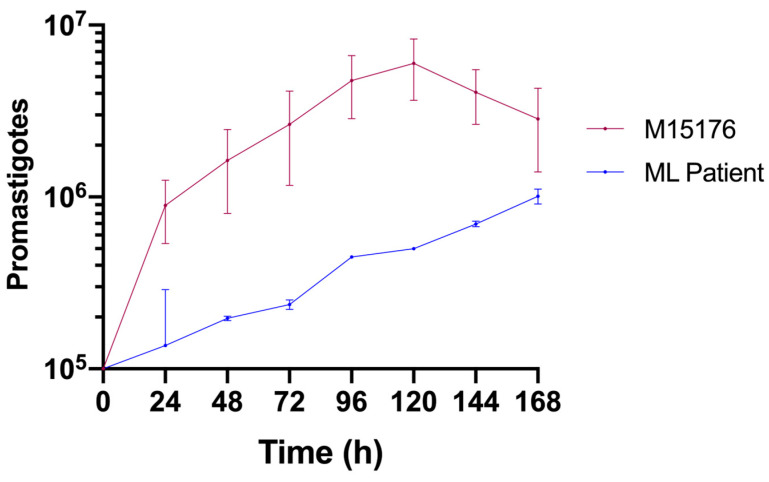
Comparative growth curves of the *L.* (*V.*) *braziliensis* control (red) and ML (blue) strains in Schneider medium supplemented with 20% FBS.

Compared with the control strain, the strain isolated from the ML patient had a lower growth rate (Figure 2). The control strain achieved the metacyclic phase on the fifth day, whereas the ML strain did not achieve this phase during the same period.

Compared with the control strain, the *Leishmania* (*V.*) *braziliensis* strain isolated from this patient presented a low proliferative capacity (Figure 2). As has already been shown in other eukaryotes [41], a subpopulation of quiescent *Leishmania* parasites can be found in persistent infections [42]. This population has been linked to drug resistance and treatment failure in murine and in vitro studies [41]. It is possible that the altered proliferative state of the parasite we studied is one of the adaptative changes that allowed it to persist in this host despite multiple treatment courses.

Results from quantitative mass spectrometry analysis showed an increased abundancy of mtHSP70 and the ATPase alpha subunit in the patient isolate compared with those in the control (Table 2).

This high level is consistent with the possibility that these proteins are associated with therapeutic failure. ATPases constitute a highly conserved family of enzymes that are responsible for ATP metabolism in prokaryotic and eukaryotic cells [43]. F ATPases present in the mitochondrial membrane of trypanosomatids catalyse both the synthesis and hydrolysis of ATP and are also called ATP synthases [43]. F ATPases play important roles in *Leishmania* metabolism and are responsible for both ATP synthesis and the maintenance of the mitochondrial membrane potential; thus, F ATPases are a theoretical target for drug development against leishmaniasis [40]. Experimental studies have not yet established the practical application of this physio-pathological knowledge to diseases caused by protozoans. On the one hand, a low expression of F ATPases could favour parasite survival in some situations. For example, one study has shown that the expression of the F1 ATPase alpha subunit is decreased in benznidazole-resistant *Trypanosoma cruzi* [44]. Similarly, in *Leishmania* (*L.*) *donovani*, an ATPase inhibitor has been shown to diminish the uptake of pentamidine by the parasite [45]. Similarly, the loss of *Leishmania* (*L.*) *amazonensis* virulence has been associated with increased expression of F ATPase [46]. On the other hand, some studies have shown that drugs that impair F ATPase function have leishmanicidal proprieties. Roy et al. reported that 3,3′-diindolylmethane (DIM), a DNA topoisomerase I inhibitor, disrupts F ATPase function in *Leishmania* spp. parasites, leading to depolarization of the mitochondrial membrane followed by programmed cell death [47]. This also seems to be the sequence of events responsible for the leishmanicidal proprieties of camptothecin [48]. Similar to what we have shown in this study, a clonal population of antimonony-resistant *Leishmania* (*L.*) *panamensis* also expresses relatively high levels of F ATPases [49]. Studies on the pathogenic potential of ATPase in *Leishmania* (*V.*) *braziliensis* are lacking, but the finding of greater production of this protein in this patient sample seems to favour the possible pathogenic role of the molecule. This result, however, may not be applicable to a wider population, and a larger study including samples from patients responsive and not responsive to treatment is warranted to reach the most definitive conclusions.

The pathogenic role of parasitic HSP70 has already been studied because this protein is highly expressed in *Leishmania* species, which are responsible for human disease [50]. It is known to promote resistance to oxidative stress but direct immunological effects through extracellular transport [51], leading to macrophage activation, and TNF-alpha production may also play a role [51]. Treatment resistance has also been associated with a specific mutation in the HSP70 gene (T579A), linking 75% of failures to antimony therapy, leading to a sevenfold increase in the risk of failure in the presence of this polymorphism (OR = 7.29; 95% CI = [1.17–45.25]; *p* = 0.0331) [52]. In a murine model, an HSP70 inhibitor used to treat leishmaniasis has shown promising results [53]. An inhibitor of the protozoan HSP70 Polymyxin B [54] has been shown to have leishmanicidal activity [55], and the possible confirmation of the role of this chaperone in parasite resistance opens the possibility of repurposing this drug, which is already commercially available, for the treatment of refractory leishmaniasis. Our study thus reinforces the potential pathogenic role of this protein and suggests its potential as a therapeutic target and as a marker for poor clinical outcomes.

Regarding our limitations, it could be argued that the multiple clinical and parasitological relapses of ML described were in fact reinfections. Importantly, however, a sterile cure of leishmaniasis is the exception rather than the rule [56], which by itself does not eliminate the possibility that the lesions are the result of a new infecting strain. However, we must also consider that after the first cutaneous ulcer, no more cutaneous lesions occurred in this case. Many authors consider that the main mechanism for parasite infection in the mucosa is through dissemination from a cutaneous lesion [57,58], and a kDNA signature study revealed that parasites from mucosal lesions are more similar to those from cutaneous lesions of the same patient than those from unrelated patients [59]; we believe that the hypothesis of reinfection in this patient is unlikely.

There is genetic variability between parasites isolated from the same patient and those from clones of the same *Leishmania* reference strain, representing a naturally mixed infection [60]. Changes in the genetic structure of one strain maintained in culture change over time in different DNA batches [61] and can be accompanied by changes in phenotype, such as loss of infectivity and the development of biochemical and antigenic changes [62]. Thus, we question whether our findings are not a result of a selection process rather than a true phenotypic difference between the strains analysed. Importantly, despite the existence of heterogeneity, genetic variability at the interpatient level is less pronounced than that between different strains [59], and different genetic profiles have been associated with phenotypic characteristics of the infecting parasite [63]. Changes in parasites over time are a limitation of in vitro studies such as ours. However, we have taken precautions to limit the modification of parasite biological and biochemical characteristics by avoiding culturing for long periods and by culturing the tested strains in mice before experiments [12,64].

## 4. Conclusions

Monitoring of this patient revealed that ML caused by *Leishmania* (*V.*) *braziliensis* might be a therapeutic challenge. In this case, both host and parasite factors seemed to be important in determining the lack of response to treatment over 25 years. The dramatic outcome observed illustrates the necessity of new drugs for this neglected disease, indicating possible new therapeutic targets. The targeting of molecules involved in the parasitic oxidative machinery or its molecular integrity is a promising strategy for future drug development. Our study reinforces the possible utility of some compounds already studied in experimental settings, and we hope that our findings lead to new studies of the possible mechanisms behind Leishmania drug resistance.

## Figures and Tables

**Table 1:** Treatments for American cutaneous leishmaniasis given to the patient for 25 years at the University Hospital of Brasilia, Brazil.

Drug	Year	Duration (Days)	Year–Dosage/Day–Duration (Days)–Evolution	Outcome
N-methyl glucamine	1987	30	20 mg SbV/kg/day	Clinical cure
	1988	20	20 mg SbV/kg/day	Clinical cure
	2002	30	20 mg SbV/kg/day	Clinical improvement
N-methyl glucamine + allopurinol	1996	30	20 mg SbV/kg/day + 15 mg/kg/day	Clinical cure
Aminosidine sulfate	1993	25	16 mg/kg/day (2 series)	Clinical cure
	1994	25	16 mg/kg/day (1 series)	Clinical cure
	1999	25	16 mg/kg/day (1 series)	Clinical improvement
Itraconazol + allopurinol	1996	15	200 mg + 15 mg/kg/day	Therapeutic failure
N-methyl glucamine + thalidomide	2000	30	20 mg SbV/kg + 200 mg/day	Clinical cure
Liposomal amphotericin B	1992	28	4050 mg cumulative dose	Clinical cure
	2002	90	2100 mg cumulative dose	Clinical improvement
N-methyl-glucamine + pentoxifylline	1998	30	20 mg SbV/kg + 1200 mg/day	Clinical improvement
Leishvacin + N-methyl glucamine	2004	130	From 0.1 mL in the first day to 1 mL in the 10th week, once per week; 15 mg SbV/kg every other day, series of 10 days with intervals of 10 days–13 weeks	Clinical improvement
Miltefosine	2007	42	2 mg/kg/day	Clinical and parasitological cure
Pentamidine	1995	48	1500 mg cumulative dose	Clinical cure
Liposomal amphotericin B + N-methyl glucamine	2005	40	2000 mg cumulative dose + 20 mg SbV/kg/day	Clinical improvement
Liposomal amphotericin B + N-+ methyl glucamine + Leishvacin	2006–2007	180	Cumulative dose 3000 mg + 20 mg SbV/kg/day-(7 series)	Clinical improvement
Miltefosine + liposomal amphotericin B + pentoxyphylline	2010	34	750 mg + 1200 mg cumulative doses. Treatment interruption due to creatinine increase	Clinical improvement
Liposomal amphotericin B + pentoxyphylline	2011/2012	180	3000 mg cumulative dose + 1200 mg/day	Clinical improvement

**Table 2:** Quantitative analysis of selected proteins’ femtomole (fmol).

Protein	*L.* (*V.*) *braziliensis* (Control)	*L.* (*V.*) *braziliensis* (Patient Isolate)
Putative heat shock protein 70-related protein 1 mitochondrial precursor *Leishmania* (*V.*) *braziliensis*	32.38	65.33
ATPase alpha subunit *Leishmania* (*V.*) *braziliensis*	30.3553	166.27

Standard: alcohol dehydrogenase (ADH) = 25 fmol.

## Data Availability

The data presented in this study are available upon request from the corresponding author due to privacy concerns.

## Supplementary Materials

The following supporting information can be downloaded at: https://www.mdpi.com/article/10.3390/biomedicines12102227/s1, Figure S1: Discriminatory sequences in the ITS1 region from the Leishmania gender; Table S1: Proteins identified in *L.(V.) braziliensis* control strain (MHOM/BR/94/M15176) by analysis carried out with the “Protein Lynx” software using the *L.(V.) braziliensis* database, from data collected by mass spectrometry of the tryptic peptides; Table S2: Proteins identified in *L.(V.) braziliensis* isolated from the patient sample by analysis carried out with the “Protein Lynx” software using the *L.(V.) braziliensis* database, from data collected by mass spectrometry of the tryptic peptides
