# Supplementary material for: Treatment of Refractory Mucosal Leishmaniasis Is Associated with Parasite Overexpression of HSP70 and ATPase and Reduced Host Hydrogen Peroxide Production (Brief Report)
Source: Biomedicines. 2024 Sep 30;12(10):2227. doi: 10.3390/biomedicines12102227 (PMC11504370; doi:10.3390/biomedicines12102227)
Supplement: Supplementary file 1 [file biomedicines-12-02227-s001.zip › Species identification.pdf]

Species identification

Genomic DNA culture leishmania was purified using the Purelink Genomic DNA Kit (Invitrogen, Waltham, MA, USA). For amplification of the internal transcribed spacer region 1 (ITS1) of ribosomal DNA, the primers PR280 (5`AGCTGGATCATTTTCCGATG-3') and PR281 (5`-TATGTGAGCCGTTATCCAGC-3') were used, which anneal to the conserved SSU and 5.8S sequences. Sequencing of the PCR product of the ITS1 region of rDNA using the ABI 3130xI sequencer(Applied Biosystems, Waltham, MA, USA). A search was carried out for Neotropical species of the subgenus Viannia that presented nucleotide sequences of the ITS1 region registered in the National Center for Biotechnology Information NCBI using the basic local alignment search tool. These nucleotide sequences were aligned in the Bioedit software to identify possible Single nucleotide polymorphism capable of distinguishing these species. We were able to distinguish the patient sample as *Leishmania (V.) braziliensis* comparing its sequence with other possible leishmania(supplementary Figure S1).

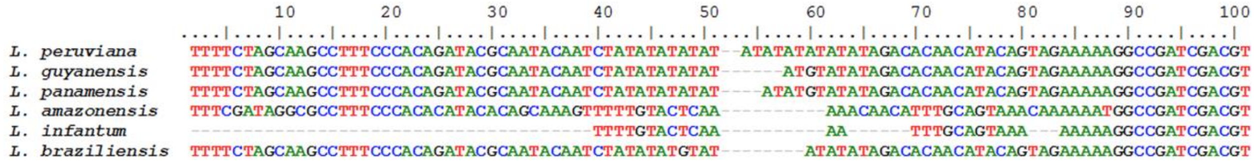


**Supplementary Figure S1**: *Discriminatory sequences in the ITS1 region from the Leishmania gender.*
