# Supplementary material for: Treatment of Refractory Mucosal Leishmaniasis Is Associated with Parasite Overexpression of HSP70 and ATPase and Reduced Host Hydrogen Peroxide Production (Brief Report)
Source: Biomedicines. 2024 Sep 30;12(10):2227. doi: 10.3390/biomedicines12102227 (PMC11504370; doi:10.3390/biomedicines12102227)
Supplement: Supplementary file 1 [file biomedicines-12-02227-s001.zip › Supplemental Tables S1 and S2.pdf]

Supplementary Table S1. Proteins identified in *L.(V.) braziliensis* control strain (MHOM/BR/94/M15176) by analysis carried out with the "Protein Lynx" software using the *L.(V.) braziliensis* database, from data collected by mass spectrometry of the tryptic peptides.

Protein	Entry	mW (Da)	pI	Peptides	Theoretical Peptides	Coverage (%)
ATPase alpha subunit *L. braziliensis*	134059187	62576	9.9697	8	40	12.1951
putative heat shock 70 related protein 1 mitochondrial precursor *L. braziliensis*	134064193	70484	5.8301	9	59	16.4363

Supplementary Table S2. Proteins identified in *L.(V.) braziliensis* isolated from the patient sample by analysis carried out with the "Protein Lynx" software using the *L.(V.) braziliensis* database, from data collected by mass spectrometry of the tryptic peptides.

Protein	Entry	mW (Da)	pI	Peptides	Theoretical Peptides	Coverage (%)
ATPase alpha subunit *L. braziliensis*	134059187	62576	9.9697	4	40	7.3171
putative heat shock 70 related protein 1 mitochondrial precursor *L. braziliensis*	134064193	70484	5.8301	13	48	19.5719
