# Supplementary material for: Treatment of Refractory Mucosal Leishmaniasis Is Associated with Parasite Overexpression of HSP70 and ATPase and Reduced Host Hydrogen Peroxide Production (Brief Report)
Source: Biomedicines. 2024 Sep 30;12(10):2227. doi: 10.3390/biomedicines12102227 (PMC11504370; doi:10.3390/biomedicines12102227)
Supplement: Supplementary file 1 [file biomedicines-12-02227-s001.zip › Supplementary digestion protocol.pdf]

Protein digestion LC- MS/MS

1. Place **50** µL of 1 µg/µL sample in a capped microcentrifuge tube.
2. Add **10** µL 50 mM NH_4HCO_3.
3. Add **25** µL of 0.2% solution of RapiGest SF and vortex. A 0.2% solution of RapiGest SF is prepared by adding 500 µL of water to a 1 mg vial of RapiGest SF (***Waters PART No. 186001861***, ***5 x 1 mg***).
4. Place tube in a block heater set at 80oC. Heat for 15 minutes.
5. Remove from block heater. Centrifuge. Add **2.5** µL of 100 mM dithiothreitol (15.4 µg/µL, DTT, for opening up the protein to make it more accessible for alkylation and digestion), vortex.
6. Place tube in a block heater set at 60oC and heat for 30 minutes.
7. Remove from block, allow cooling to room temperature, centrifuge.
8. Add **2.5** µL of 300 mM iodoacetamide (55.5 µg/µL, IAA, for alkylation of cysteines), vortex.
9. Place sample in dark at room temperature and allow 30 minutes reaction time.
10. Add **10** µL of a solution of Madison Trypsin in 50 mM NH_4HCO_3 (add 400 µL of 50 mM NH_4HCO_3 to one 20 µg vial of ***Promega Trypsin, PART No. V511A, 100 µg in 5 x 20 µg aliquots***, lyophilized), vortex. Digest at 37oC in a block heater overnight. This is a 1:100 wt:wt ratio of enzyme:protein.
11. Following the digestion, to hydrolyze the RapiGest, add **10** µL of 5% TFA (prepared from high purity ampoules, ***Pierce, PART No. 53102, HPLC Grade, 10 x 1 mL***), and vortex. Incubate samples at 37°C for 90 minutes. Then centrifuge the samples at 14,000 RPM, 6°C for 30 minutes. Transfer the supernatant to a Waters Total Recovery vial (***Waters PART No. 186000385c, 100/pkg, preslit PTFE/silicone caps***).
12. Add **5** uL of 1 pmol/uL ADH (***MassPREP Digestion Standard Alcohol Dehydrogenase, PART No. 186002328, 1 nmol/vial***. To prepare 1 pmol/µL ADH, add 1 mL of 3% ACN, 0.1% FA to one vial.) Then add **85** uL of 3% ACN, 0.1% FA. The final concentration of protein is 250 ng/uL, and of ADH is 25 fmol/uL. The final volume is 200 µL.
